# Gait initiation and termination strategies in patients with Prader-Willi syndrome

**DOI:** 10.1186/s12984-017-0257-7

**Published:** 2017-05-23

**Authors:** Veronica Cimolin, Nicola Cau, Manuela Galli, Cristina Santovito, Graziano Grugni, Paolo Capodaglio

**Affiliations:** 1 Department of Electronics, Information and Bioengineering, Politecnico di Milano, Piazza Leonardo da Vinci, 32, 20133 Milan, Italy; 2 IRCCS “San Raffaele Pisana”, Tosinvest Sanità, Rome, Italy; 3 Orthopaedic Rehabilitation Unit and Clinical Lab for Gait Analysis and Posture, Ospedale San Giuseppe, Istituto Auxologico Italiano, IRCCS, Via Cadorna 90, I-28824 Piancavallo (VB), Italy; 4 Unit of Auxology, Ospedale San Giuseppe, Istituto Auxologico Italiano, IRCCS, Via Cadorna 90, I-28824 Piancavallo (VB), Italy

**Keywords:** Gait initiation, Gait termination, Prader-Willi syndrome, Obesity, Rehabilitation, Center of pressure

## Abstract

**Background:**

Gait Initiation (GI) is a functional task representing one of the first voluntary destabilizing behaviours observed in the development of a locomotor pattern as the whole body centre of mass transitions from a large to a small base of support. Conversely, Gait Termination (GT) consists in the transition from walking to standing which, in everyday life, is a very common movement. Compared to normal walking, it requires higher control of postural stability. For a safe GT, the forward movement of the body has to be slowed down to achieve a stable upright position. Stability requirements have to be fulfilled for safe GT. In individuals with Prader-Willi syndrome (PWS), excessive body weight negatively affects the movement, such as walking and posture, but there are no experimental studies about GI and GT in these individuals. The aim of this study was to quantitatively characterise the strategy of patients with PWS during GI and GT using parameters obtained by the Center of Pressure (CoP) track.

**Methods:**

Twelve patients with PWS, 20 obese (OG) and 19 healthy individuals (HG) were tested using a force platform during the GI and GT tasks. CoP plots were divided into different phases, and duration, length and velocity of the CoP trace in these phases were calculated and compared for each task.

**Results:**

As for GI, the results showed a significant reduction of the task duration and lower velocity and CoP length parameters in PWS, compared to OG and HG. In PWS, those parameters were reduced to a higher degree with respect to the OG. During GT, longer durations, similar to OG, were observed in PWS than HG. Velocity is reduced when compared to OG and HG, especially in medio-lateral direction and in the terminal part of GT.

**Conclusions:**

From these data, GI appears to be a demanding task in most of its sub-phases for PWS individuals, while GT seems to require caution only towards the end of the task. Breaking the cycle of gait into the phases of GI and GT and implementing specific exercises focusing on weight transfer and foot clearance during the transition phase from the steady condition to gait will possibly improve the effectiveness of rehabilitation and fall and injury prevention

## Background

There is now a body of literature on various “sub-tasks” of walking that may compromise stability, such as initiation, termination, turning, obstacle crossing, negotiating a raised surface and stair climbing. In all these tasks balance is challenged during the transition from one, either statically stable or dynamically stable movement pattern, to another [1]. However, limited focus has been placed on gait initiation and termination. The dynamic processes of gait initiation and termination are much more complex since the human body needs to accelerate and decelerate, respectively, often in a limited amount of time. As a result, the skills necessary to maintain stability, weight transfer, foot clearance, etc., become more critical during these transition phases than during the steady state conditions [2, 3]. Such requirements become even more significant in patients with neurological disorders, lower limb complications, and in older adults, where there are inherent difficulties with postural stability and gait [4, 5].

In particular, gait initiation (GI) represents the transition from standing to walking, it is a task that is often required in daily life and challenges balance control [6, 7]. Compared to steady-state walking, the requirements on the neuromuscular system are increased in GI, since a complex integration of neural mechanisms, muscle activity and biomechanical forces is necessary [6]. GI is a functional task representing one of the first voluntary destabilizing behaviours observed in the development of a locomotor pattern as the whole body centre of mass (COM) transitions from a large to small base of support (from a bipedal to a monopedal position related to gait). This task represents a challenge to the postural control system due to the volitional transition from a condition of relatively static stable support to one of continuously unstable posture during locomotion [8–11] and one that has been shown to be a sensitive indicator of balance dysfunction [11].

Conversely, gait termination (GT) consist in the transition from walking to standing that, in everyday life, is a very common movement [12]. Compared to normal walking, it requires higher control of postural stability and a complex integration and cooperation within the neuromuscular system [6, 13, 14]. For a safe GT, the forward movement of the body has to be slowed down to achieve a stable upright position [13, 15]. Stability requirements have to be fulfilled for safe gait termination. In the final bipedal standing position, the COM coincides with the Centre of Pressure (CoP) and lies within the base of support [16, 17].

These two motor tasks have been studied to provide insight into dynamic postural control and the changes that occur in the control system only with advancing age and disability, such as in individual with Parkinson, individual with multiple sclerosis, obeses and individuals with lower limb amputation [1, 7, 9, 18–28]. To the best of our knowledge, no studies on GI and GT in patients with Prader-Willi syndrome (PWS) have been published so far. Prader-Willi syndrome (PWS) is the most frequent cause of syndromic obesity with an estimated prevalence of 1:10.000/30.000 [29]. Its major clinical features include muscular hypotonia, childhood-onset obesity, short stature, small hands and feet, scoliosis, osteoporosis, hypogonadism and developmental delays [30]. Typically, PWS patients present with reduced lean body mass and increased fat to lean mass ratio not only when compared with lean patients but also in relation to obese patients [31, 32]. Obesity and excessive amounts of fat modify the body geometry by adding passive mass to different regions and influence the biomechanics of activities of daily living, causing functional limitations, and possibly predisposing the patient to injury. Quantitative evidence exists that excessive body weight negatively affects the movement from sitting to standing and walking in obese [33] and in PWS individuals [34]. Body mass increases can also be a major factor contributing to the occurrence of falls, which explains why obese persons appear to be at greater risk than normal-weight individuals under daily postural stresses and perturbations [35, 36].

According to these considerations, patients with PWS may cope with more difficulty with GI and GT demands. Previous papers have addressed the poor balance [37, 38] and gait difficulties [34, 39] in individuals with PWS, unveiling the motor abnormalities related both to an excessive body mass under static and dynamic conditions and to some dysmorphic features, as short stature, small hands and feet, scoliosis and muscular hypotonia. Given that GI and GT could be destabilizing activities for PWS patients, the purpose of this study was to quantitatively characterise the strategy of these individuals during these tasks using parameters obtained by the Center of Pressure (CoP) track. The results were compared with those obtained in a group of non-genetically obese subjects and in a group of normal weight subjects.

## Methods

### Participants

In this study, 3 groups of participants were recruited. Twelve adult patients (7 females, 5 males; age: 36.6 ± 6.6 years; height: 1.57 ± 0.04 m; BMI: 38.1 ± 6.9 kg/m^2^) with a diagnosis of PWS were enrolled in this study. The PWS patients had been periodically hospitalized at the San Giuseppe Hospital, Istituto Auxologico Italiano, Piancavallo (Verbania), Italy. During hospital stay, patients undergo a 4-week multidisciplinary rehabilitation program. At admission, a thorough clinical assessment was performed. All patients showed the typical PWS clinical phenotype Hass [40]. Cytogenetic analysis was performed in all patients; 11 out of them had interstitial deletion of the proximal long arm of chromosome 15 (del15q11-q13). Uniparental maternal disomy for chromosome 15 (UPD15) was found in one female. All PWS patients showed mild mental retardation. In this respect, one of the requirements for participating in the study was a score over the cut-off value of 24 in the Mini Mental State Examination (MMSE) Italian version [41]. Scores over the MMSE cut-off are recognized as suggesting the absence of widespread acquired cognitive disorders in adult people. Our PWS patients were all able to understand and complete the testing.

Two different reference groups of subjects were specifically recruited for this study and served as controls. The first group included 20 obese patients (Obese Group: OG; 13 females, 7 males; age: 40.7 ± 11.7 years; height: 1.67 ± 0.1 m; BMI: 41.9 ± 4.0 kg/m^2^). This group was selected in order to have a BMI match group. Exclusion criteria were the presence of neurological disorders, oculo-vestibular disorders, major musculo-skeletal condition (complicated back, hip and knee pain, flat foot), hip and knee replacements, and arrhythmia. Inclusion criteria was the ability to walk independently without aids.

The second group consisted of 19 age-matched healthy subjects (Healthy Group: HG; 11 females, 8 males; age: 33.9 ± 11.2 years; height: 1.72 ± 0.1 m; BMI: 21.2 ± 1.3 kg/m^2^) recruited among the hospital staff. Inclusion criteria for the HG were no cardiovascular, neurological or musculoskeletal disorders. They had normal flexibility and muscle strength and no obvious gait abnormalities. All participants had normal values in the main laboratory tests, including adrenal and thyroid function. All participants were able to walk independently without aids. The study was approved by the Ethics Committee of the Institute; written informed consent was obtained by the patients.

### Experimental setup

The study was performed in the motion analysis laboratory of S Giuseppe Hospital, which is equipped with a 8-m-long walkway and 2 force platforms (Kistler, Winterthur, Switzerland) in order to measure the trajectory of CoP. In this study, only one force platform was used.

For the GI task, subjects were asked to stand barefoot in a relaxed posture, on a bipedal standing position on the force platform with feet in a fixed and parallel position (the distance between their heels was fixed to the pelvis width). Acquisition of force platform data was triggered just prior to the participants receiving a verbal cue to begin walking- approximately 3 s before starting task. In response to the cue, the participants initiated walking; the leading limb stepped off the force platform first, followed by the trailing limb and continued walking for several steps. All the requests were standardized: 3 trials starting with the left foot and 3 trials starting with the right foot. For each subject the gait velocity was self-selected [26, 27].

For the GT task, subjects were asked to walk at their self-selected velocity, to stop walking on the force platform with both legs and to stand still for at least 3 s. The participants terminate walking by stepping with the leading limb on the force plate, followed by placing the trailing limb next to the leading limb. They performed at least three steps prior to the GT step, to achieve the steady-state gait [25]. Adjustment of the step length in order to hit the force plate was avoided by practicing the task in advance to select an appropriate distance from the starting point to the force plate. The subjects were instructed to look at the end of the walkway instead of at the force plate. All the requests were standardized: 3 trials stopping with the left foot and 3 with the right foot. Experimental sessions took place at the beginning of the rehabilitation program for all subjects.

### Data analysis

The raw CoP data sampled at a frequency of 1 kHz and low-pass-filtered at 20 Hz was analysed using a dedicate protocol developed using SmartAnalyzer software (version: 1.10.451.0; BTS, Italy).

**Fig. 1:**
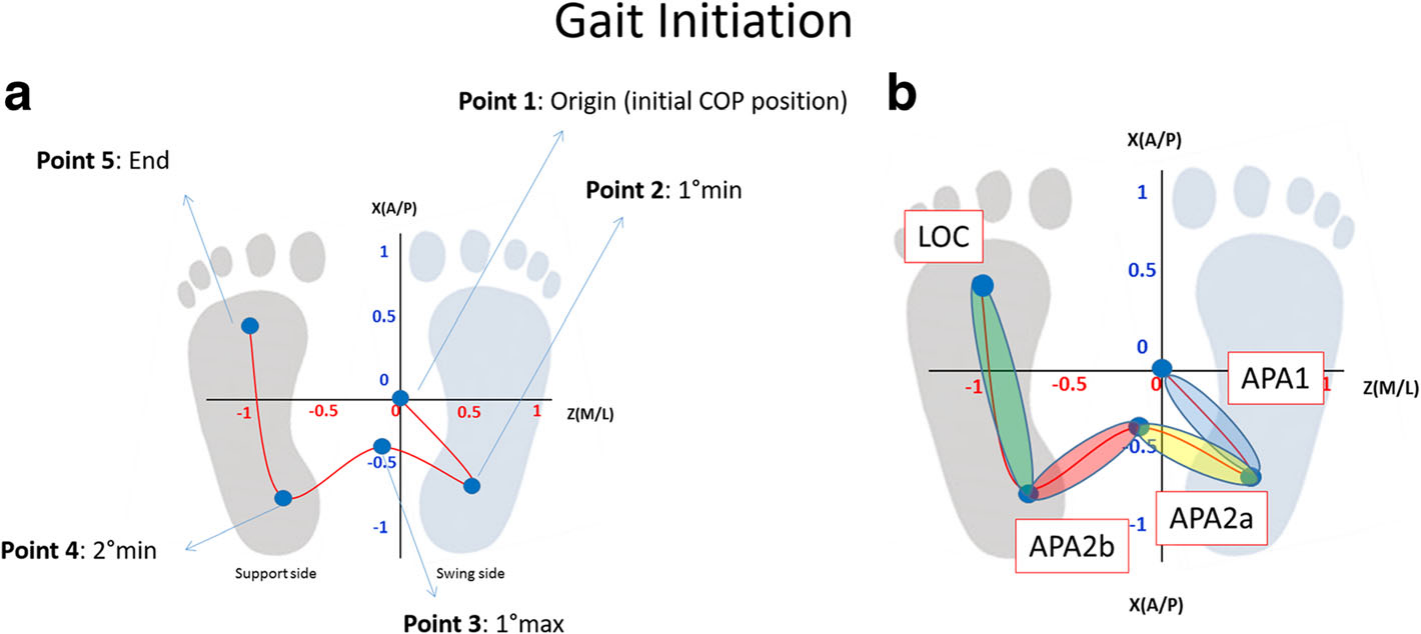
**a** Overhead view of Centre of Pressure (COP) displacement in antero-posterior and medio-lateral direction during Gait Initiation (GI). For this task the support sides is the *left* foot and the swing side is the *right* foot. **b** Centre of Pressure (COP) trajectory division for the analysis during GI (APA1, APA2a, APA2b, LOC)

For each acquisition of GI task, five points were manually identified as shown in Fig. 1a:

1. Origin (initial CoP position)
2. First minimum (1 min): minimum posterior position of the CoP on the leg in swing side
3. First maximum (1max): Maximum anterior position during the CoP transition from the leg in swing to the leg in stance
4. Second minimum (2 min) minimum posterior position of the CoP on the leg in stance side.
5. End (Final CoP position).

For the timing analysis, we divided the task in two phases [7, 24, 27] as shown in Fig. 1b:

1. postural phase, which is computed between a quite standing position and the start of the task. This first phase of GI - typically referred to as an anticipatory postural adjustment (APA). It can be divided into two sub-phases:
  APA1 begins at the onset of the movement and ends at the release of swing foot vertical loading - this APA is between the origin and the first minimum. It represents the translation of the CoP in lateral and posterior directions together toward the swing foot heel
  APA2 that begins at swing foot release, ends at the swing toe off, and represents a lateral CoP shift toward the stance foot [7]. APA2 was further divided into two additional sub-phases: APA2a and APA2b defined respectively the anticipatory movement between the first minimum and the first maximum and the anticipatory movement between the first maximum and the second minimum
2. locomotor phase - following referred to as LOC - which is between the second minimum and the end of the COP trajectory.

**Fig. 2:**
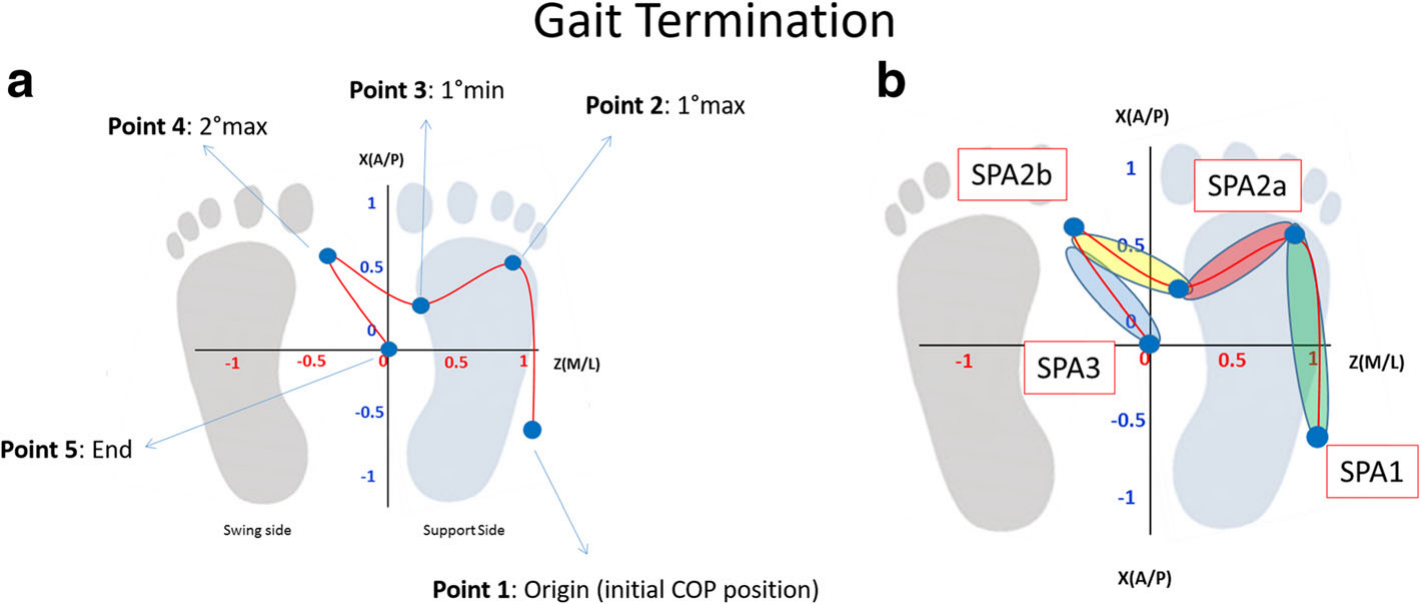
**a** Overhead view of Centre of Pressure (COP) displacement in antero-posterior and medio-lateral direction during Gait Termination (GT). For this task the support sides is the *right* foot and the swing side is the *left* foot (**b**) Centre of Pressure (COP) trajectory division for the analysis during GT (SPA1, SPA2a, SPA2b, SPA3)

For each acquisition of GT task, five points were manually identified - as shown in Fig. 2a:

1. Origin (initial CoP position)
2. First maximum (1max): maximum anterior position of the CoP on the leg in swing side
3. First minimum (1 min): minimum posterior position during the CoP transition from the leg in swing to the leg in stance
4. Second maximum (2max) maximum anterior position of the CoP on the leg in stance side.
5. End (Final CoP position).

For the timing analysis, we divided the GT task in two phases [1, 25] - as shown in Fig. 2b:

1. locomotor phase - following referred to as SPS 1 (Stopping Postural Adjustment - SPA) - which is between the initial CoP origin to the first maximum of the CoP trajectory
2. postural phase, which is computed between the end of the locomotor phase and the quite standing position.

This phase can be divided into two sub-phases - Fig. 2b:

- SPA 2 that begins at the end of the LOC phase and ends just prior the initial contact of the swing limb. SPA 2 was further divided into two additional sub-phases: SPA 2b and SPA 2a defined respectively the anticipatory movement between the first maximum and the minimum and the anticipatory movement between the minimum and the second maximum
- SPA3 begins at the initial contact of the swing limb and ends at the final bipedal stance position-this phase is between the second maximum and the final CoP position.

According to these phases subdivision, the following parameters have been calculated for both tasks:

- ▪ Track duration (s) during each segment (as for GI: TAPA1, TAPA2A, TAPA2B, TOLOC; as for GT: TSPA1, TSPA2A, TSPA2B, TSPA3), and the total duration of the task (TTOT as for GI and GT) (expressed as the sum of the duration of each segment);
- ▪ Track velocity (m/s) in the segments and in the anterior-posterior direction (x) and in medio lateral direction (z) (as for GI: VAPA1 (x,z), VAPA2A (x, z), VAPA2B (x, z), VLOC (x, z); as for GT: VSPA 3(x,z), VSPA 2a(x,z), VSPA 2b(x,z), VSPA 1(x,z));
- ▪ Track length (mm/m) of each segment (as for GI: LAPA1, LAPA2A, LAPA2B, LLOC; as for GT: LSPA3, LSPA2a, LSPA2b, LSPA1) [29]. All track length parameters were normalized by subjects’ height (m).

Statistical analysis was conducted using Statistica (version 7.0; StatSoft Inc, USA). All parameters were computed bilaterally for each participant and the median and quartile range values of all indexes were calculated for each group (PWS, OG and HG). Kolmogorov-Smirnov tests were used to verify if the parameters were normally distributed; the parameters were not normally distributed, so we used the Kruskal-Wallis tests followed the post hoc analysis for comparing data among all three groups. Level of significance was set at *p* < 0.05.

## Results

### Gait initiation

In the starting posture, no differences were found as for feet position in all of the evaluated subjects; we therefore pooled the data from both sides. The values (median and quartile range) of parameters related to GI are displayed in Table 1 for all the evaluated groups. All time durations (TAPA1, TAPA2A, TAPA2B, TLOC and TTOT parameters) were higher in the PWS and OG than HG. The PWS and OG showed lower values of velocities than HG in the anterior/posterior direction (VAPA1x, VAP2Ax, VAP2Bx and VLOCx); in addition, VAPA1x, VAP2Ax and VLOCx were statistically different between PWS and OG, with PWS showing lower values than OG. The velocities in the medial/lateral direction during the APA1 (VAPA1z index) and APA2A phase (VAPA2z index) were statistically lower in PWS with respect to the OG and HG. No statistically differences were displayed in VAP2Bz and VLOCz parameters among the three evaluated groups. The PWS showed statistically lower values than OG and HG of some track lengths (LAPA1, LLOC and LTOT parameters). The LAPA2A length was lower in PWS than OG, but not with respect to HG: PWS showed in fact similar values compared to HG. No differences among groups were found in LAPA2B length.

**Table 1:** Values of median and quartile range for the calculated parameters during Gait Initiation task

GAIT INITIATION			
	PWS	OG	HG
*DURATION [s]*			
TAPA1	0.45 (0.21)*	0.44 (0.19) *	0.32 (0.14)
TAPA2A	0.18 (0.08)*	0.17 (0.07) *	0.13 (0.08)
TAPA2B	0.13 (0.06)*	0.13 (0.06)*	0.11 (0.06)
TLOC	0.54 (0.11)*	0.56 (0.11) *	0.50 (0.08)
TTOT	1.34 (0.33)*	1.35 (0.27)*	1.09 (0.23)
*VELOCITY [m/s]*			
VAPA1_(x)_	0.05 (0.04)*+	0.08 (0.07)*	0.15 (0.12)
VAPA2A_(x)_	0.07 (0.07)*+	0.10 (0.08)*	0.14 (0.10)
VAPA2B_(x)_	0.09 (0.08)*	0.12 (0.10)*	0.18 (0.11)
VLOC_(x)_	0.22 (0.06)*+	0.24 (0.10)*	0.29 (0.10)
VAPA1_(z)_	0.05 (0.05)*+	0.09 (0.07)	0.08 (0.08)
VAPA2A_(z)_	0.29 (0.18)*+	0.39 (0.26)	0.39 (0.24)
VAPA2B_(z)_	0.35 (0.23)	0.42 (0.20)	0.39 (0.23)
VLOC_(z)_	0.03 (0.05)	0.03 (0.03)	0.02 (0.03)
*LENGTH [mm/m]*			
LAPA1	0.029 (0.010)*+	0.041 (0.019)	0.040 (0.019)
LAPA2A	0.037 (0.018)+	0.052 (0.029) *	0.035 (0.019)
LAPA2B	0.036 (0.018)	0.032 (0.026)	0.028 0.025)
LLOC	0.080 (0.011)*+	0.093 (0.023)	0.092 (0.016)
LTOT	0.180 (0.035)*+	0.216 (0.052)*	0.192 (0.039)

PWS (Prader-Willi Syndrome group), OG (Obese Group) and HG (Healthy Group). * = *p* < 0.05, PWS and/or OG Vs. HG;, + = *p* < 0.05, PWS Vs. OG

### Gait termination

In the final posture, no differences were found in feet position in all of the evaluated subjects; we therefore pooled the data from both sides. The values (median and quartile range) of parameters related to GT are displayed in Table 2 for all the evaluated groups. The PWS and OG showed higher values than HG of some duration parameters (TSPA3, TSPA2A and TTOT indexes); in addition, TSPA3 was higher in PWS in comparison to OG. As for the velocities, PWS were characterised by lower values of VSPA1x, VSPA3z and VSPA2Az if compared to HG and OG, with the exclusion of VSPA1x, which was similar between PWS and OG. Regarding length parameters, no differences were found between PWS and OG with in general similar values respect to HG. The LSPA2A value was higher in PWS and OG if compared to HG.

**Table 2:** Values of median and quartile range for the calculated parameters during Gait Termination

GAIT TERMINATION			
	PWS	OG	HG
*DURATION [s]*			
TSPA1	0.40 (0.08)	0.46 (0.13)	0.43 (0.11)
TSPA2A	0.19 (0.16)*	0.19 (0.15)*	0.14 (0.09)
TSPA2B	0.17 (0.10)	0.13 (0.11)	0.15 (0.13)
TSPA3	0.55 (0.12)*+	0.50 (0.22)*	0.39 (0.05)
TTOT	1.37 (0.25)*	1.29 (0.40)*	1.12 (0.23)
*VELOCITY [m/s]*			
VSPA 1_(x)_	0.26 (0.15)*	0.29 (0.12)*	0.37 (0.12)
VSPA2A_(x)_	0.06 (0.07)	0.07 (0.07)	0.07 (0.09)
VSPA2B_(x)_	0.08 (0.07)	0.06 (0.09)	0.10 (0.07)
VSPA3_(x)_	0.03 (0.04)	0.03 (0.04)	0.03 (0.03)
VSPA1 _(z)_	0.05 (0.09)	0.06 (0.09)	0.05 (0.09)
VSPA2A_(z)_	0.19 (0.15)*+	0.27 (0.23)	0.25 (0.25)
VSPA2B_(z)_	0.33 (0.21)	0.33 (0.38)	0.27 (0.21)
VSPA3_(z)_	0.02 (0.03)*+	0.04 (0.05)	0.04 (0.06)
*LENGHT [mm/m]*			
LSPA1	0.083 (0.038)	0.088 (0.033)	0.095 (0.033)
LSPA2A	0.031 (0.018)*	0.029 (0.031)*	0.025 (0.023)
LSPA2B	0.034 (0.013)	0.032 (0.029)	0.030 (0.017)
LSPA3	0.025 (0.019)	0.028 (0.019)*	0.022 (0.014)
LTOT	0.175 (0.068)	0.180 (0.053)	0.175 (0.034)

PWS (Prader-Willi Syndrome group), OG (Obese Group) and HG (Healthy Group). * = *p* < 0.05, PWS and/or OG Vs. HG;, + = *p* < 0.05, PWS Vs. OG

**Fig. 3:**
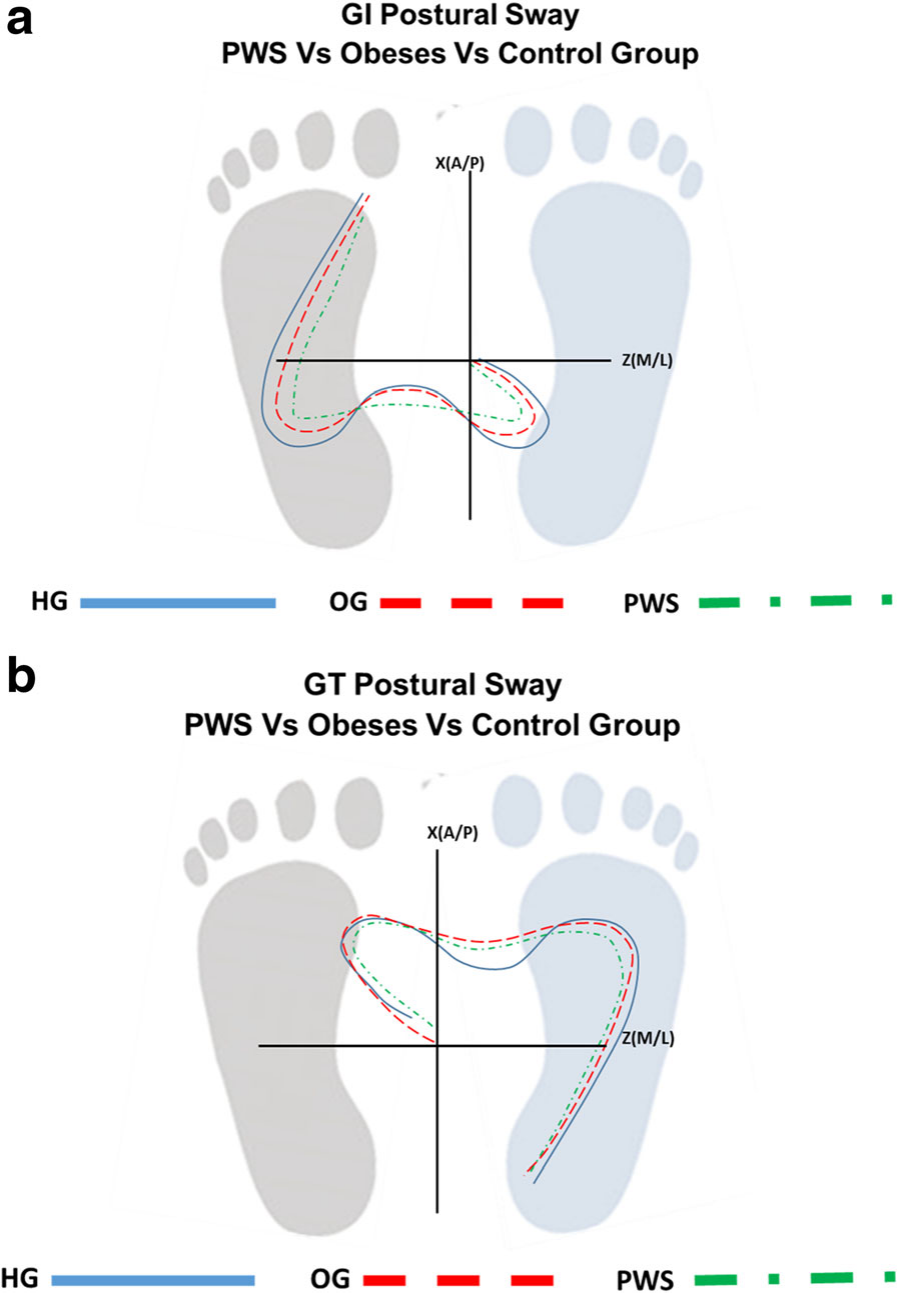
A representative comparison between the postural sway path of an individual representative of PWS group, one of the OG and one of the HG during GI task (**a**) and during the GT task (**b**)

A representative figure of the postural sway during both tasks and between the three groups is showed in Fig. 3.

## Discussion

GI, the transition from standing to walking, and GT, from walking to standing, may represent destabilizing activities for PWS patients. In this study, we aimed to quantitatively compare the GI and GT patterns of adult PWS individuals with those observed in non-genetically obese and in lean individuals. Due to the common occurrence of mental retardation, however, it could be argued that cognitive impairment may have a significant influence on the values observed in PWS. Nevertheless, the inclusion criterion of a score >24 in the MMSE, a reliable method to rule out a serious deterioration of mental functions, seems to exclude this negative effect.

As for GI, the results showed a significant reduction of velocity and CoP length parameters in PWS, with respect to the non-genetically obese subjects. The excessive body weight of the PWS leads, together with a reduced muscle strength and motor control, characteristic features of these individuals [32, 42], to a further reduction of the velocity and length parameters during most of APAs phases, as compared to OG and HG [27]. The role of the adductor hip muscles is fundamental for GI; in fact, these muscles allow the shifting of CoP from one side to the contralateral [26]. The results obtained in this study could be related to a reduction of hip adductor activity in the PWS patients. Short feet and height, which are common features of PWS patients, may have also accounted for the reduction in CoP length. However, to exclude it, the CoP length parameters were presented after normalization to the patients’ height. As lower normalized values were observed in PWS, we can speculate that reduced muscle strength and motor control in PWS as compared to the non-genetically obese population may have had a significant impact on reduction in CoP length.

Longer durations, similar to OG, were observed in PWS than HG during GT. Velocity is reduced when compared to OG and HG, especially in medial-lateral direction and in the terminal part of GT (SPA2A and SPA3). We can speculate that this strategy may be used by PWS patients in order to achieve better stability while terminating gait. These results could be related not only to the excessive body mass but also to the decreased motor control of PWS [37]. It appears therefore likely that they use a more cautious strategy and a slower pace at the end of GT.

From our experimental data, GI appears to be a demanding task in most of its sub-phases (APA1, APA2A and LOC) for PWS individuals, while GT seems to require caution only towards the end of the task. It appears even more so that the combination of high body mass, reduced motor control and muscle strength could account for PWS difficulties in negotiating GI. The difference in the results could be related to the more complexity of GI. In GI APAs contribute to postural stability and to create the propulsive forces necessary to reach steady state gait at a predefined velocity and may be indicative of the effectiveness of the feedforward control of gait. Our findings could be related to an abnormal muscular activation pattern mainly characterized by a disruption of the synergistic activity of antagonistic pairs of postural muscles and they suggest that individuals with PWS might lack accurate tuning of feedforward control of movements at GI. In addition, the practice of the task for GT could influence this difference.

## Conclusions

Possible rehabilitative spin-offs of this study include the implementation within the rehabilitation program of specific exercises to improve stability and motor control. In particular, breaking the cycle of gait into the phases of GI and GT and implementing specific exercises focusing on weight transfer and foot clearance during the transition phase from the steady condition to gait will possibly improve the effectiveness of rehabilitation and fall and injury prevention. In addition, balance exercises, eye/feet coordination, walking on different surfaces and crossing obstacles are some of the goals in motor rehabilitation.

The limitations of this study were the followings: firstly, only data related to CoP trajectory were investigated while no evaluations of lower limb joints kinematics and kinetics were conducted. As it represents the first attempt to quantify GI and GT strategies of individuals with PWS, we decided to perform the assessment using only one force platform, with a less time-consuming evaluation than a thorough 3D investigation including markers placement. Secondly, the small number of participants resulting in limited strength of the statistical findings. We have to bear in mind, however, that PWS is a rare genetic condition and large experimental samples are difficult to gather. However, it is important to underline that, despite these limitations, this represents the first study focusing on GI and GT performance in PWS subjects.

## Abbreviations

APA: Anticipatory postural adjustment
CoP: Centre of Pressure
GI: Gait Initiation
GT: Gait termination
LOC: Locomotor phase
PWS: Prader-Willi syndrome
T: Time
TOT: Total
